# *Laminaria japonica* Aresch-Derived Fucoidan Ameliorates Hyperlipidemia by Upregulating LXRs and Suppressing SREBPs

**DOI:** 10.1155/2024/8649365

**Published:** 2024-02-12

**Authors:** Yan Zhang, Tian Liu, Ze-Jie Qu, Xue Wang, Wen-Gang Song, Shou-Dong Guo

**Affiliations:** ^1^Department of Endocrinology and Metabolism, Guiqian International General Hospital, Guiyang 550018, China; ^2^Institute of Lipid Metabolism and Atherosclerosis, Innovative Drug Research Centre, School of Pharmacy, Weifang Medical University, Weifang 261053, China; ^3^Cardiology Department, Qingzhou People's Hospital, Weifang 262500, China; ^4^Shandong Provincial Key Laboratory for Rheumatic Disease and Translational Medicine, The First Affiliated Hospital of Shandong First Medical University & Shandong Provincial Qianfoshan Hospital, Jinan 250014, China

## Abstract

Cardiovascular disease (CVD) is the leading cause of morbidity and mortality worldwide, and hyperlipidemia is one major inducing factor of CVD. It is worthy to note that fucoidans are reported to have hypolipidemic activity with species specificity; however, the underlying mechanisms of action are far from clarification. This study is aimed at investigating the plasma lipid-lowering mechanisms of the fucoidan from *L. japonica* Aresch by detecting the levels of hepatic genes that are involved in lipid metabolism. Our results demonstrated that the fucoidan F3 significantly lowered total cholesterol and triglyceride in C57BL/6J mice fed a high-fat diet. In the mouse liver, fucoidan F3 intervention significantly increased the gene expression of peroxisome proliferator-activated receptor (PPAR) *α*, liver X receptor (LXR) *α* and *β*, and ATP-binding cassette transporter (ABC) G1 and G8 and decreased the expression of proprotein convertase subtilisin/kexin type 9 (PCSK9), low-density lipoprotein receptor, cholesterol 7 alpha-hydroxylase A1, and sterol regulatory element-binding protein (SREBP) 1c and SREBP-2. These results demonstrated that the antihyperlipidemic effects of fucoidan F3 are related to its activation of PPAR*α* and LXR/ABC signaling pathways and inactivation of SREBPs. In conclusion, fucoidan F3 may be explored as a potential compound for prevention or treatment of lipid disorders.

## 1. Introduction

Cardiovascular disease (CVD) is a leading cause of morbidity and mortality worldwide, and it is induced by distinct factors including hyperlipidemia. However, the clinical hypolipidemic drugs could not completely retard the progression of CVD [1–3]. It is worth noting that fucoidans are reported to possess various biological effects *in vitro* and *in vivo* such as anticoagulant, antithrombotic, anticancer, anti-inflammatory, antiviral, antioxidant, hepatoprotective, immunomodulatory, neuroprotective, and gut microbiota-modulatory functions [4–9].

Importantly, fucoidans are reported to have a hypolipidemic activity [10, 11]. For instance, fucoidans obtained from *Sargassum henslowianum* reduce total cholesterol (TC), triglyceride (TG), and low-density lipoprotein cholesterol (LDL-c) in albino house mice of BALB/c strain fed a high-fat diet [12]. Another study demonstrates that fucoidan from *Fucus vesiculosus* (family Fucaceae) reduces TC, TG, and LDL-c and increases high-density lipoprotein cholesterol (HDL-c) in poloxamer-407-induced hyperlipidemic C57BL/6 NtacSam mice. The underlying mechanisms are related to upregulation of liver low-density lipoprotein receptor (LDLR) and downregulation of sterol regulatory element-binding protein (SREBP) 2 [13]. The fucoidan derived from *Cladosiphon okamuranus* also reduces TC, TG, and non-HDL-c and increases HDL-c and lipoprotein lipase (LPL) activity in apolipoprotein E-deficient mice by activating the expression of peroxisome proliferator-activated receptor (PPAR) *α* mRNA and inactivating SREBP1 mRNA [14]. Low-molecular-weight fucoidan obtained from *C. okamuranus* reduces TG and LDL-c and shows no significant effect on HDL-c in diet-induced obese SD rats with surgery-induced osteoarthritis [15]. Furthermore, the fucoidan from sea cucumber *Acaudina molpadioides* exhibits antiadipogenic activity by downregulating the expression of SREBP-1c mRNA in high-fat diet mice [16], and sea cucumber *Pearsonothuria graeffei*-derived fucoidan reduces TC, LDL-c, and fat tissue weight in C57BL/6 mice fed a high-fat diet [17]. More importantly, fucoidan administration significantly reduces LDL-c in obese adults [18].

The plasma lipid-lowering mechanisms of fucoidans may differ along with their origin [19]. *Laminaria japonica* is a kind of brown alga, and it is widely cultivated in Asia, especially in China. In 2010, fucoidans obtained from *L. japonica* Aresch were found to decrease serum levels of TC, TG, and LDL-c in hyperlipidemic rats and increase the level of serum HDL-c and the activities of LPL, hepatic lipoprotein, and lecithin cholesterol acyltransferase [20]. However, their mechanisms of action are still far from clear. Reverse cholesterol transport (RCT) is believed to facilitate hyperlipidemia, and this mechanism plays a key role in cholesterol homeostasis [21]. In this study, we report for the first time that fucoidan from *L. japonica* Aresch may exert its hypolipidemic effects by modulating the expression of RCT-related genes in C57 BL/6J mice fed a high-fat diet.

## 2. Materials and Methods

### 2.1. Materials

Crude fucoidan of *L. japonica* (also known as *Saccharide japonica*) was prepared by Weihai Rensheng Pharmaceutical Group Co., Ltd. in the year of 2018 (Weihai, China). 1-Phenyl-3-methyl-5-pyrazolone (PMP), monosaccharide standards, and dextran standards were obtained from Sigma-Aldrich (St. Louis, MO, USA). Fenofibrate was bought from Macklin (purity 99%, Shanghai, China). A BCA assay kit and dialysis membranes were purchased from Solarbio (Beijing, China). KBr powder was bought from Tianjin Guangfu Technology Development Co., Ltd. (Tianjin, China). TC and TG assay kits were the products of Biosino Bio-technology and Science Inc. (Beijing, China). All reagents used in this study were of analytical grade.

### 2.2. Deproteinization and Separation of the Fucoidan

The deproteinization reagent was prepared by mixing chloroform and n-butanol at a ratio of 4 : 1 (*v*/*v*). Crude fucoidan solution (50.0 mg/mL) was mixed with freshly prepared deproteinization reagent at a ratio of 5 : 1. After shaking for 10 min, the mixture was centrifugated at 5,000 × *g* for 20 min at room temperature. The above processes were repeated twice. Next, the supernatant was collected and dialyzed against tap water (molecular weight, Mw cutoff 8,000~14,000 Da) for 2 days and distilled water for 1 day. Next, the fucoidan solution in the dialysis membrane bag was freeze-dried.

The deproteinized samples were separated according to our previously reported method [22]. In brief, the Q-Sepharose™ Fast Flow column loaded with crude fucoidan was washed using 0-2 mol/L NaCl solution. The obtained carbohydrates were monitored using the phenol-sulfuric acid method [23]. In this study, the fraction named as F3 was further separated via a Sephacryl S200HR column. The detailed method has been documented in the literature [22].

### 2.3. Chemical Analysis of the Fucoidan F3

Total sugar content, sulfate ester content, uronic acid content, and protein content were determined by phenol-sulfuric acid [23], BaCl_2_-gelatin method [24], carbazole-sulfuric acid method [25], and a BCA assay kit, respectively. Monosaccharide composition was detected using high performance liquid chromatography (HPLC) after precolumn derivatization by PMP according to a previous literature [26]. Fourier-transform infrared spectrometry (FTIR) analysis was performed using a Nicolet iS5 spectrometer as previously described methods [23, 26].

### 2.4. Animals

Twenty C57BL/6J mice (male, 23.5 ± 1.5 g) were randomly divided into vehicle group (0.9% sodium chloride, vehicle), fenofibrate group (50 mg/kg/d), low-dose F3 group (50 mg/kg/d, F3-L), and high-dose F3 group (200 mg/kg/d, F3-H) with five mice in each cage. This experiment was approved by the Laboratory Animal Ethical Committee of Weifang Medical University and followed the NIH Guidelines for the Care and Use of Animals. All mice were fed a high-fat diet (21% fat, 0.15% cholesterol, 20% protein, and 34% sucrose) that was bought from Beijing HFK Bioscience Co., Ltd. (license number: SCXK2014-0008). Compounds were given by intragastric administration, and the dosage was determined by previous literatures [13, 17, 23]. The mice were allowed to free access to food and water during the 6-week experimental period. It has been demonstrated that a period of 6 weeks is enough to develop hyperlipidemia and obesity in mice or rats [15, 17].

### 2.5. Plasma Analysis

Animals were sampled after overnight fasting, and the plasma levels of TC and TG were determined using commercially available assay kits.

### 2.6. Real-Time Quantitative PCR

Total RNA was prepared using TRIzol reagents (SparkJade, Qingdao, China) according to the manufacturer's instructions. The concentration and purity of the RNA were determined using a UV spectrophotometer. In general, the total RNA with an absorbance ratio of 260/280 greater than 1.90 was used for the following studies. The reverse transcription of cDNA and real-time PCR was performed according to the previous literature [22]. The primers are listed in Table 1. The relative amount of target mRNA was calculated by the comparative cycle threshold (*C*_t_) method according to the 2^-*ΔΔ*Ct^ formula.

### 2.7. Data Analysis

Results were expressed as mean ± standard deviation (SD) for at least three independent experiments. Statistical analysis was performed using the Student *T*-test. Differences were considered to be significant at a *P* < 0.05.

## 3. Results

### 3.1. Purification and Chemical Analysis of Fucoidan F3

**Figure 1:**
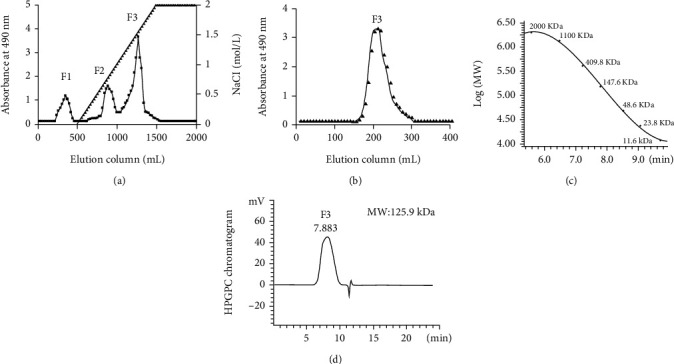
Preparation of fucoidan F3: (a) the crude fucoidan was loaded onto a Q-Sepharose™ Fast Flow column (5.0 × 30 cm) and eluted with 0.0-2.0 mol/L NaCl; (b) the fraction F3 was separated with a Sephacryl S200HR column (2.6 × 90 cm) using 0.2 mol/L NH_4_HCO_3_ as the eluent; (c) the standard curve of dextran standards; (d) the chromatogram and Mw of fucoidan F3.

**Figure 2:**
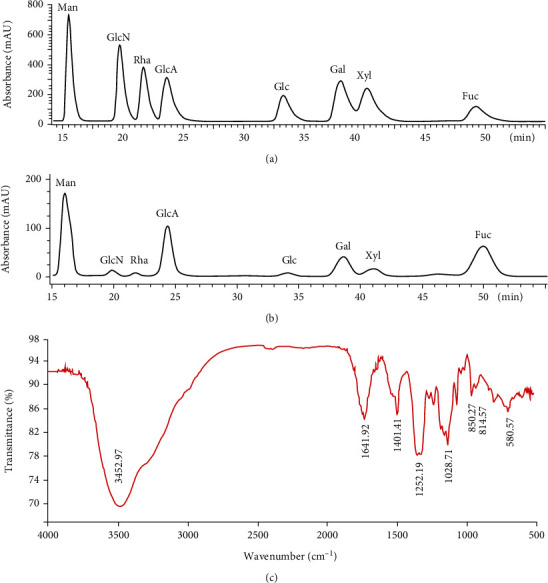
Chemical characteristics of fucoidan F3: (a) the standard curve of eight monosaccharides in an equal molar ratio as detected by HPLC and the detection wavelength was 245 nm; (b) monosaccharide composition of fucoidan F3; (c) FTIR spectrum of fucoidan F3. Man: D-mannose; GlcN: D-glucosamine; Rha: L-rhamnose; GlcA: D-glucuronic acid; Glu: D-glucose; Gal: D-galactose; Xyl: D-xylose; Fuc: L-fucose.

As shown in Figure 1(a), three fractions were obtained from the Q-Sepharose™ Fast Flow column after linear elution by 0-2 mol/L of NaCl. F3 with the greatest yield of 47.9% was used for the following study. This fraction appeared as a single peak on the Sephacryl S200HR column (Figure 1(b)). Its average Mw was calculated as 125.9 kDa (Figures 1(c)and 1(d)) according to the curve made by dextran standards. The carbohydrate and sulfate contents of F3 were 52.6% and 22.8%, respectively. The content of protein and uronic acid was 0.1% and 12.3%, respectively. HPLC analysis demonstrated that F3 was mainly consisted of mannose, glucuronic acid, galactose, xylose, and fucose in a molar ratio of 1.0 : 1.3 : 1.2 : 0.9 : 5.5 (Figures 2(a) and 2(b)). The dominant fucose took ~55.6% of the total sugar content. Fucoidan F3 also contained minor glucosamine, rhamnose, and glucose as shown in Figure 2(b); however, their contents were less than 3% according to their peak area percentage.

### 3.2. FTIR Analysis

As shown in the FTIR spectrum (Figure 2(c)), the broad and strong signals at 3452.97 cm^−1^ and 1095.31 cm^−1^ were due to the stretching vibration and bending vibration of hydroxyl groups (-OH), respectively [26]. The bands at 1401.41 cm^−1^ were assigned to the bending vibration of alkyl groups (-CH_2_- and –CH_3_). The signals at approximately 1028.71 cm^−1^ were attributed to the stretching vibrations of C-O-C and C-O-H. The signals at 1252.19 cm^−1^ were attributed to the asymmetric stretching vibration of the sulfate esters (O=S=O). Furthermore, the bands at 814.57-850.27 cm^−1^ were originated from C-O-S bending vibration of the sulfate substitute, both at the axial C2 and the equatorial C4 positions [22].

### 3.3. F3 Alleviated Obesity and Hyperlipidemia in C57BL/6J Mice

**Figure 3:**
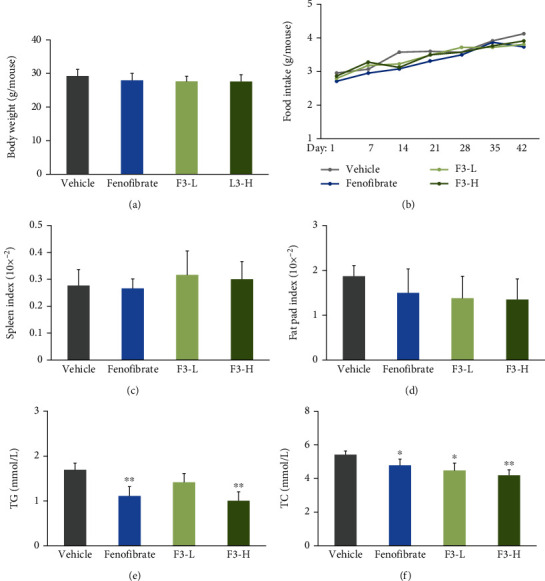
Effects of fucoidan F3 in hyperlipidemic C57BL/6J mice (*n* = 5): (a) average body weight; (b) average food intake; (c) spleen index; (d) fat pad index; (e) plasma TG level; (f) plasma TC level. F3-L: 50 mg/kg/d of fucoidan F3; F3-H: 200 mg/kg/d of fucoidan F3. ^∗^*P* < 0.05 vs. vehicle group; ^∗∗^*P* < 0.01 vs. vehicle group. The abbreviations are suitable for the rest of the figures.

As shown in Figures 3(a) and 3(d), the body weight and fat pad index of the mice showed no significant difference among groups. These data were consistent with the results of food intake among groups (Figure 3(b)). Furthermore, F3 investigation did not affect the spleen index (Figure 3(c)). Importantly, F3 intervention significantly decreased plasma TG level by approximately 41% at the dosage of 200 mg/kg, and the effect was close to that of fenofibrate (*P* < 0.01, Figure 3(e)). Moreover, F3 reduced plasma TC level in a dose-dependent manner (*P* < 0.01 in F3-H group). The TC-lowering effect in the F3-H group was greater than that of the fenofibrate group (Figure 3(f)). These results indicated that fucoidan F3 was effective on inhibiting high-fat diet-induced hyperlipidemia in C57BL/6J mice.

### 3.4. Effects of Fucoidan F3 on the Gene Expression of SREBP-2, LDLR, PCSK9, SR-B1, PPAR*α*, and PPAR*γ* in Mouse Liver

**Figure 4:**
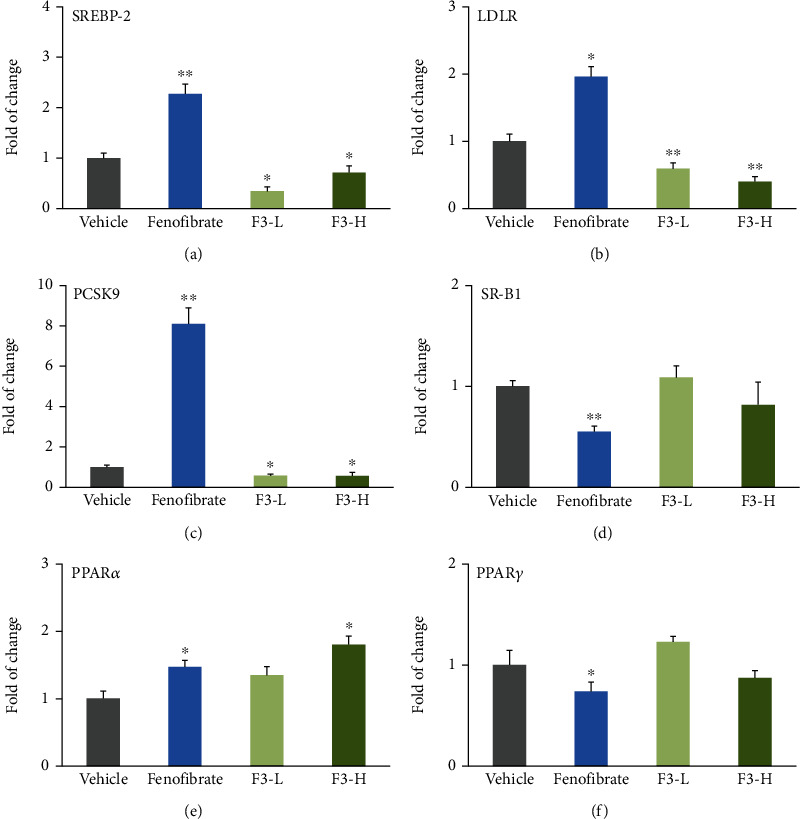
Effects of fucoidan F3 on gene expression of (a) SREBP-2, (b) LDLR, (c) PCSK9, (d) SR-B1, (e) PPAR*α*, and (f) PPAR*γ* in mouse liver (*n* = 3). ^∗^*P* < 0.05 vs. vehicle group; ^∗∗^*P* < 0.01 vs. vehicle group.

SREBP-2 regulates cholesterol homeostasis by upregulating cholesterol synthesis and hepatic uptake of circulating non-HDL particles through LDLR. As shown in Figures 4(a)–4(c), fenofibrate treatment significantly increased the gene expression of SREBP-2 by approximately 1.3-fold (*P* < 0.01, Figure 4(a)) and dramatically enhanced the downstream genes LDLR (*P* < 0.05, Figure 4(b)) and PCSK9 (*P* < 0.001, Figure 4(c)) compared to the vehicle group. It is worth noting that fenofibrate increased the gene expression of PCSK9 by ~7.1-fold. On the contrary, fucoidan F3 intervention significantly reduced the gene expression of SREBP-2 by approximately 65% and 30% in the F3-L and F3-H groups, respectively (*P* < 0.05, Figure 4(a)). In line with this reduction, fucoidan F3 intervention decreased the gene expression of LDLR in a dose-dependent manner (Figure 4(b)) and significantly reduced the gene expression of PCSK9 by approximately 45% (*P* < 0.01, Figure 4(c)). These data suggested that fucoidan F3 showed contrary effects on LDLR and PCSK9 mRNA expression compared to that of fenofibrate. As shown in Figure 4(d), fenofibrate intervention significantly decreased the gene expression of SR-B1 by approximately 42% (*P* < 0.01), and fucoidan F3 did not significantly affect the expression of this gene at both low and high dosage groups.

In the present study, fenofibrate significantly increased the gene expression of PPAR*α* by approximately 47% (*P* < 0.05, Figure 4(e)) and dramatically decreased the gene expression of PPAR*γ* by ~26% (*P* < 0.01, Figure 4(f)). More importantly, fucoidan F3 administration improved the gene expression of PPAR*α* in a dose-dependent manner and exhibited significant difference at 200 mg/kg compared to the vehicle group (*P* < 0.01, Figure 4(e)). Unlike fenofibrate administration, fucoidan F3 did not affect the gene expression of PPAR*γ* (Figure 4(f)). Therefore, fucoidan F3 showed different effects on modulation of PPAR genes.

### 3.5. Effects of Fucoidan F3 on the Gene Expression of LXR*α*, LXR*β*, ABCG1, ABCG8, CYP7A1, and SREBP-1c in Mouse Liver

**Figure 5:**
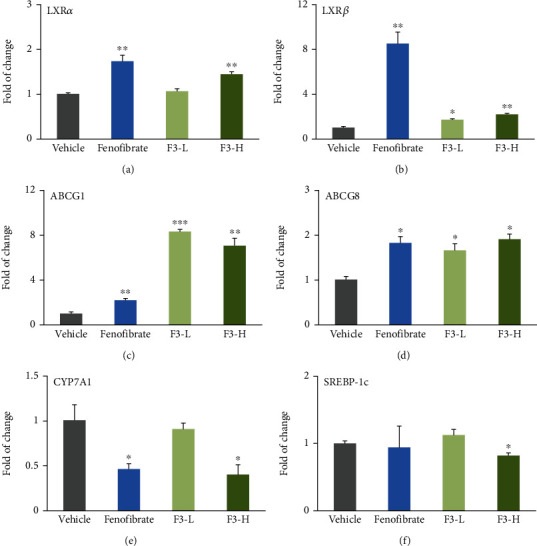
Effects of fucoidan F3 on gene expression of (a) LXR*α*, (b) LXR*β*, (c) ABCG1, (d) ABCG8, (e) CYP7A1, and (f) SREBP-1c in mouse liver (*n* = 3). ^∗^*P* < 0.05 vs. vehicle group; ^∗∗^*P* < 0.01 vs. vehicle group; ^∗∗∗^*P* < 0.001 vs. vehicle group.

Fenofibrate treatment significantly elevated the gene expression of LXR*α* and LXR*β* by approximately 73% and 7.5-fold, respectively, compared to the vehicle group (*P* < 0.01, Figures 5(a) and 5(b)). Furthermore, fenofibrate significantly enhanced the downstream genes of LXRs. It improved the mRNA expression of ABCG1 and ABCG8 by approximately 1.2-fold (*P* < 0.01) and 82% (*P* < 0.05), respectively (Figures 5(c) and 5(d)). Interestingly, fucoidan F3 administration significantly improved the mRNA expression of LXR*α* by approximately 44% in the F3-H group (*P* < 0.05, Figure 5(a)) and elevated LXR*β* mRNA expression in a dose-dependent manner (Figure 5(b)). It is worth noting that F3 intervention increased the gene expression of LXR*β* by approximately 69% (*P* < 0.05) and 1.2-fold (*P* < 0.01) in the F3-L and F3-H groups, respectively. Interestingly, fucoidan F3 administration improved the gene expression of ABCG1 by approximately 7.3-fold (*P* < 0.001) and 6.1-fold (*P* < 0.01), respectively, compared to the vehicle group (Figure 5(c)). Furthermore, fucoidan F3 administration also significantly increased the gene expression of ABCG8 by approximately 65% and 90% in the F3-L and F3-H groups, respectively (Figure 5(d)). Like fenofibrate, fucoidan F3 administration dramatically decreased the gene expression of CYP7A1 by approximately 54% at the dosage of 200 mg/kg (Figure 5(e), *P* < 0.05). Of importance, fucoidan F3 intervention significantly reduced the gene expression of SREBP-1c by approximately 18% (Figure 5(f), *P* < 0.05). However, fenofibrate treatment did not affect the expression of SREBP-1c in the present study.

## 4. Discussion

Accumulating evidence has demonstrated that antihyperlipidemic compounds can alleviate CVDs [1–3]. Previous publications have indicated that fucoidans have plasma lipid-lowering effects with species specificity; however, the underlying mechanisms of action are still far from clarification [10–13]. This study investigated the plasma lipid-lowering mechanisms of action of the brown seaweed *L. japonica* Aresch-derived fucoidan F3 by evaluating the expression of multiple genes involved in lipid metabolism in the liver of C57BL/6J mice fed a high-fat diet. These innovative data demonstrated that this fucoidan F3 may (1) ameliorate hypercholesterolemia by elevating LXR/ABC signaling pathways and suppressing the gene expression of SREBP-2 and (2) improve hypertriglyceridemia by activating PPAR*α* without modulating the gene expression of PPAR*γ* and decreasing the gene expression of SREBP-1c.

The monosaccharide composition of fucoidan F3 was close to that of seaweed *C. okamuranus*-derived fucoidan (36.2% fucose, 6.7% glucuronic acid) made by a Japan company [14]. However, the purity of deproteinized and purified fucoidan F3 should be greater than that of *C. okamuranus*-derived fucoidan made by the Oriental Bio. Co., Ltd. [14]. In this study, we found that the fucoidan F3 significantly reduced the plasma TC and TG. These results were consistent with the effects of fucoidans from seaweed *C. okamuranus* and *F. vesiculosus* and sea cucumber *A. molpadioides* [13, 14, 16]. However, several other studies found the fucoidans derived from seaweed *C. okamuranus* and *S. henslowianum* and sea cucumber *P. graeffei* have no effects on TG [12, 15, 17]. These differences may be induced by the differences in preparation of fucoidan as well as the animal models. For instance, seaweed *C. okamuranus*-derived fucoidans were prepared by different companies in the references [14, 15]. Furthermore, the F-fucoidan, a product of Swanson Health (Fargo, ND, USA), showed on effects on TG at a dose of 500 mg/d in overweight or obese adults [18]. It seems that fucoidans, at the dosage around 100 mg/kg/d, may reduce TC levels by approximately 20% in rodents mainly through reduction of LDL-c as some fucoidans are reported to improve HDL-c levels [12–15, 17]. Another study demonstrated that the TC-lowering effects of *C. okamuranus*-derived fucoidan increased from 21.8% to 29.6% as its dosage increased from 32 mg/kg to 320 mg/kg [15]. The liver is an important organ for lipid metabolism. To elucidate the plasma lipid-lowering mechanism of fucoidan F3, we investigated 12 genes that are involved in lipid metabolism in the present study. Among these genes, ABC transporters, LXRs, PPARs, SR-B1, LDLR, and PCSK9, are RCT-related genes.

There are three distinct PPAR subfamily members that are encoded by distinct genes. PPAR*α* is highly expressed in the liver and is the molecular target of a series of fibrates used for treatment of hypertriglyceridemia. Mechanism studies indicated that PPAR*α* directly regulates a network of genes encoding proteins that are required for uptake of fatty acids and enzymes that are required for fatty acid oxidation (*β*-oxidation) [27]. PPAR*γ* is a master transcriptional regulator of adipogenesis and plays a key role in the process of lipid storage [27]. Therefore, PPAR*α* and PPAR*γ* have opposing effects on regulation of fat metabolism. As a PPAR*α* agonist, fenofibrate activated the mRNA expression of PPAR*α* and inactivated PPAR*γ* in this study. However, this molecule may enhance the expression of PPAR*γ* in other animal models [28]. It is worth noting that fucoidan F3 showed greater effects on improving PPAR*α* gene expression at 200 mg/kg/d compared to fenofibrate (50 mg/kg/d) without significantly influencing the gene expression of PPAR*γ*. Seaweed *C. okamuranus*-derived fucoidan also increased the gene expression of PPAR*α* as well as several other genes involved in fatty acid *β*-oxidation [14]. Interestingly, sea cucumber *A. molpadioides*-derived fucoidan mainly composed of ⟶3)-*α*-D-Fuc-(1 ⟶ glycosyls (Mw 1614.1 kDa) can effectively inhibit the proliferation and differentiation of 3T3-L1 cells, a kind of preadipocyte, at a dosage of 200 *μ*g/mL [16].

SREBPs are important transcription factors involved in regulation of lipid metabolism and homeostasis in the liver. The SREBP family consists of three members: SREBP-1a, SREBP-1c, and SREBP-2. They are encoded by SREBP1 and SREBP2 genes and have different roles in lipid metabolism [29, 30]. SREBP-1c regulates the fatty acid and TG syntheses. Importantly, in line with sea cucumber *A. molpadioides*-derived fucoidan Am-Fuc, fucoidan F3 also reduced the gene expression of SREBP-1c, a key adipogenic gene involved in TG synthesis [16]. Therefore, the TG-lowering effects of fucoidan F3 may be partially attributed to its upregulation of PPAR*α* and downregulation of SREBP-1c. Several previous studies also demonstrated that *C. okamuranus*- or *L. japonica* Aresch-derived fucoidan might reduce serum TG through enhancing the activity of LPL [14, 20]. The antiadipogenic function of fucoidan has also been demonstrated to be associated with its inhibition of CCAAT/enhancer binding protein-*α* [16].

SREBP-2 specifically modulates the genes involved in cholesterol synthesis and uptake, such as 3-hydroxy-3-methylglutaryl-coenzyme A reductase (HMGCR) and LDLR, thereby modulates cholesterol synthesis and hepatic uptake [29]. The result of fenofibrate on SREBP-2 was consistent with a previous report, which demonstrated that fibrates (fenofibrate, bezafibrate, and gemfibrozil) can significantly raise the gene expression of SREBP-2 in patients [31]. However, another study indicated that fenofibrate suppresses hepatic gene expression of SREBP-2 as well as its downstream gene HMGCR in hamsters [32]. It seems that fibrates may show distinct effects based on the animal models. In the present study, fenofibrate intervention also dramatically raised the expression of SREBP-2 downstream genes including LDLR and PCSK9. It is obvious that the increase multiple of PCSK9 gene was obviously greater than that of LDLR. It is known that PCSK9 binds to the epidermal growth factor-like repeat A domain of the LDLR, inducing LDLR degradation [33, 34]. Therefore, the increased PCSK9 may decrease the actual effects of hepatic uptake of circulating LDL particles through LDLR in fenofibrate-treated mice. On the contrary, fucoidan F3 significantly decreased the gene expression of SREBP-2 and its downstream genes, LDLR and PCSK9. In line with our data, seaweed *F. vesiculosus*-derived fucoidan (a product of Sigma-Aldrich) suppresses the expression of mature SREBP-2 protein and HMGCR protein; however, this molecule significantly elevated the expression of LDLR protein [13]. These data suggested that fucoidan may decrease cholesterol synthesis by inhibiting SREBP-2 and increase hepatic uptake of LDL particles through upregulation of LDLR. The upregulation of LDLR may be attributed to the suppression of PCSK9 and other unknown translational and posttranslational mechanisms. However, our previous study demonstrated that seaweed *Ascophyllum nodosum*-derived fucoidans may enhance the mRNA expression of LDLR (A3) and improve LDLR protein (A2) at the dosage of 50 mg/kg/d [23, 35]. Therefore, fucoidans with different origin may show distinct effects on LDLR expression. The detailed mechanisms of action need to be investigated by comparative studies as well as structure-activity relationship research.

SR-B1 mediates HDL particles transfer from plasma to the liver for metabolism [36]. As the plasma profile of mice is HDL-c dominant, SR-B1 signaling pathway plays a key role in clearance of circulating HDL-c. However, fenofibrate significantly decreased the gene expression of SR-B1 in the present study. This data was consistent with a previous report which indicated that fenofibrate reduces the expression of hepatic SR-B1 protein [35]. Importantly, this data may partially explain why fucoidan F3 showed greater effect on lowering plasma cholesterol compared to fenofibrate in this study. It is worth noting that fenofibrate exhibits different effects in different cells. For instance, this molecule is found to enhance SR-B1 expression in aortic macrophages [37]. In this study, fucoidan F3 showed no significant influence on the gene expression of SR-B1. Therefore, *L. japonica* Aresch-derived fucoidan may have distinct effects on SR-B1-mediated hepatic uptake of HDL particles compared to seaweed *A. nodosum*-derived fucoidans (A2 and A3) and *C. okamuranus*-derived fucoidan (48 kDa, purity of ~90%, sulfate 14%, Kanehide Bio Co., Ltd., Okinawa, Japan), which significantly raise the expression of SR-B1 in foam cells [23, 35, 38].

The cholesterol in the liver can be converted to bile acid by a series of enzymes, and CYP7A1 is the first and rate-limiting enzyme in this process [39]. However, our results indicated that fucoidan F3 significantly suppressed the gene expression of CYP7A1 as that of fenofibrate. This result was consistent with the effects of commercially available seaweed *C. okamuranus*-derived fucoidan and inconsistent with that of *A. nodosum*-derived fucoidan A3 [23, 38]. Therefore, fucoidan with different origin may have distinct effects on the expression of CYP7A1. It is worth noting that fucoidan F3 significantly enhanced the gene expression of LXR*α* and LXR*β*, which are critical regulators of genes involved in lipid metabolism including ABC transporters, CYP7A1, and SREBP-1c [40]. It is presumed that some unknown mechanisms, such as microRNA or long-chain noncoding RNAs, are involved in modulation of the gene expression of CYP7A1 and SREBP-1c. Interestingly, fucoidan F3 dramatically raised the gene expression of ABC transporters. It is known that hepatic AGCG5/8 play key roles in hepatic lipid excretion [22, 40]. To our best knowledge, we report for the first time that *L. japonica* Aresch-derived fucoidans may exert their TC-lowering effects through activation of LXRs/ABCG8 in mouse liver. As both fenofibrate and fucoidan F3 increased the gene expression of LXRs and PPAR*α*, the greater effect of fucoidan F3 on ABCG1 mRNA compared to fenofibrate intervention may be attributed to the downregulation of PPAR*γ* by fenofibrate because PPAR*γ* has also been demonstrated to directly activate ABCG1 expression [27]. It is acknowledged that ABCG1 plays a key role in mediating cholesterol efflux from peripheral cells to mature HDL particles [41]. As a limitation of this study, we only determined the effects of fucoidan F3 on the gene expression of ABCG1 in mouse liver rather than in peripheral cells, such as macrophages. However, we presume that fucoidan F3 may also work in peripheral cells. In line with this speculation, some studies have demonstrated that fucoidan may enhance HDL-c levels in mice [13, 14]. However, this speculation needs to be investigated in the future.

## 5. Conclusion

Fucoidan F3 obtained from brown seaweed *L. japonica* Aresch exhibited powerful plasma lipid-lowering activity in C57 BL/6J mice fed a high-fat diet. The mechanisms of action are associated with the upregulation of hepatic PPAR*α* and LXR/ABC transporters and downregulation of SREBP-2, PCSK9, and SREBP-1c. However, there are limitations in this study. First of all, as structure of fucoidan determines its bioactivity, it is important to clarify the structural characteristics of this sulfated polysaccharide. Secondly, whether this fucoidan improves lipid metabolism by modulating gut microbiota needs to be investigated in the future. Thirdly, fucoidan may reduce cholesterol absorption and increase cholesterol excretion in the small intestine, and how F3 modulate the genes involved in lipid metabolism in intestine also needs to be determined in the following studies. The last but not least, it is interesting to investigate whether this molecule could be used as a nanoparticle material for targeted delivery of lipid-lowering drugs [42]. Taking together, fucoidan F3 has a great potential for prevention and treatment of dyslipidemia.

## Figures and Tables

**Table 1:** The primers used for polymerase chain reaction (PCR) reaction.

Primer	Sequences (5′–3′)
GAPDH	
Forward	AGGTCGGTGTGAACGGATTTG
Reverse	GGGGTCGTTGATGGCAACA
LXR*β*	
Forward	ATGTCTTCCCCCACAAGTTCT
Reverse	GACCACGATGTAGGCAGAGC
PPAR*γ*	
Forward	TCGCTGATGCACTGCCTATG
Reverse	GAGAGGTCCACAGAGCTGATT
PCSK9	
Forward	GAGACCCAGAGGCTACAGATT
Reverse	AATGTACTCCACATGGGGCAA
CYP7A1	
Forward	GGGATTGCTGTGGTAGTGAGC
Reverse	GGTATGGAATCAACCCGTTGTC
ABCG8	
Forward	CTGTGGAATGGGACTGTACTTC
Reverse	GTTGGACTGACCACTGTAGGT
ABCG1	
Forward	GCTCCATCGTCTGTACCATCC
Reverse	ACGCATTGTCCTTGACTTAGG
SRB1	
Forward	TGTACTGCCTAACATCTTGGTCC
Reverse	ACTGTGCGGTTCATAAAAGCA
PPAR*α*	
Forward	AACATCGAGTGTCGAATATGTGG
Reverse	CCGAATAGTTCGCCGAAAGAA
LXR*α*	
Forward	CTCAATGCCTGATGTTTCTCCT
Reverse	TCCAACCCTATCCCTAAAGCAA
LDLR	
Forward	TCGCCCTGCTCCTGGCTGCTG
Reverse	CTAGCGATGCATTTTCCGTCT
SREBP-2	
Forward	GCAGCAACGGGACCATTCT
Reverse	CCCCATGACTAAGTCCTTCAACT
SREBP-1c	
Forward	TGGACGAGCTGGCCTTCGGT
Reverse	GGCCAGCGGCAGGCTAGATG

## Data Availability

Data supporting the study results can be provided on requirement.
